# An Atypical Presentation of Post-herpetic Neuralgia and Bell’s Palsy in a Diabetic Patient: A Case Report

**DOI:** 10.7759/cureus.83014

**Published:** 2025-04-25

**Authors:** John Acosta-Peñaloza, Osman Mahboob, Natalia Kabat, Yusuf Amawi, Cynthia Tie

**Affiliations:** 1 Clinical Sciences, Florida State University College of Medicine, Tallahassee, USA; 2 Dermatology, Family Dermatology of North Florida, Tallahassee, USA

**Keywords:** bells palsy, notalgia paresthetica, post herpetic neuralgia, pruritus, varicella-zoster virus

## Abstract

Post-herpetic neuralgia (PHN) is a severe and often persistent complication of varicella-zoster virus (VZV) reactivation, particularly affecting the elderly and immunocompromised. We present the case of a 54-year-old male with a history of childhood chickenpox and type 2 diabetes mellitus who developed PHN following VZV reactivation. Initial symptoms of burning and painful paresthesia in the shoulders, combined with the subsequent onset of Bell's palsy, confirmed the diagnosis of PHN. Beginning with an initial treatment of gabapentin, significant relief was ultimately achieved with valacyclovir, emphasizing the importance of early and targeted intervention in PHN management. This case underscores the complexities of diagnosing PHN, especially in the presence of comorbidities, and highlights the need for further research to refine treatment strategies for this debilitating condition.

## Introduction

Post-herpetic neuralgia (PHN), a neuropathic pain syndrome, is a distressing and often persistent complication resulting from the reactivation of the varicella-zoster virus (VZV) [1]. Affecting elderly and immunocompromised patients primarily, PHN manifests as a severe, unrelenting pain along the affected dermatome distribution, persisting for months or even years after the initial herpes zoster (HZ) rash has resolved [2]. The pathogenesis of PHN is multifaceted, involving a complex interplay of viral-induced nerve damage, inflammatory processes, and central nervous system sensitization, posing significant challenges in diagnosis and management [3].

Current theories suggest that viral replication within the dorsal root ganglia leads to neuronal injury and subsequent aberrant neuropathic pain signaling, contributing to the development of PHN [4]. Additionally, the immune response triggered by the viral infection may perpetuate ongoing inflammation and neuropathic pain [5]. Accurate diagnosis of PHN relies on a comprehensive clinical evaluation, including a detailed medical history, physical examination, and specialized diagnostic tests, as distinguishing PHN from other neuropathic pain disorders is crucial for tailoring appropriate therapeutic interventions [6]. We discuss a case of PHN following VZV reactivation, accompanied by a thorough analysis of the clinical presentation, diagnostic approach, and management strategies. Our objective is to contribute to the existing literature, enhancing the understanding of this debilitating condition and its multifaceted implications, ultimately aiming to improve patient outcomes and quality of life.

## Case presentation

A 54-year-old male presented with paresthesia that had initially appeared on his right shoulder four months prior and subsequently spread to his left shoulder. The paresthesia was characterized by burning, itching, and painful sensations, described by the patient as "pins and needles." His medical history included childhood chickenpox and type 2 diabetes mellitus. Family history was notable for maternal shingles. Surgical history included percutaneous transluminal coronary angioplasty. The patient had no history of cutaneous shingles and had not received the shingles vaccine. Current medications included glimepiride 1 mg orally once daily and semaglutide 0.5 mg (2 mg/3 mL) injected subcutaneously once weekly for four weeks. 

The initial presentation of the paresthesia prompted the patient to seek care in the emergency department, where he was prescribed gabapentin 300 mg orally twice daily and 5% lidocaine topical patches. While lidocaine patches provided temporary symptomatic relief, gabapentin use did not lead to any significant improvement in symptoms. This prompted the patient to seek consultation at our outpatient facility. During the outpatient presentation, the patient reported a pain intensity of 7/10 on the left shoulder, with diminished severity of pain over the right shoulder.

**Figure 1:**
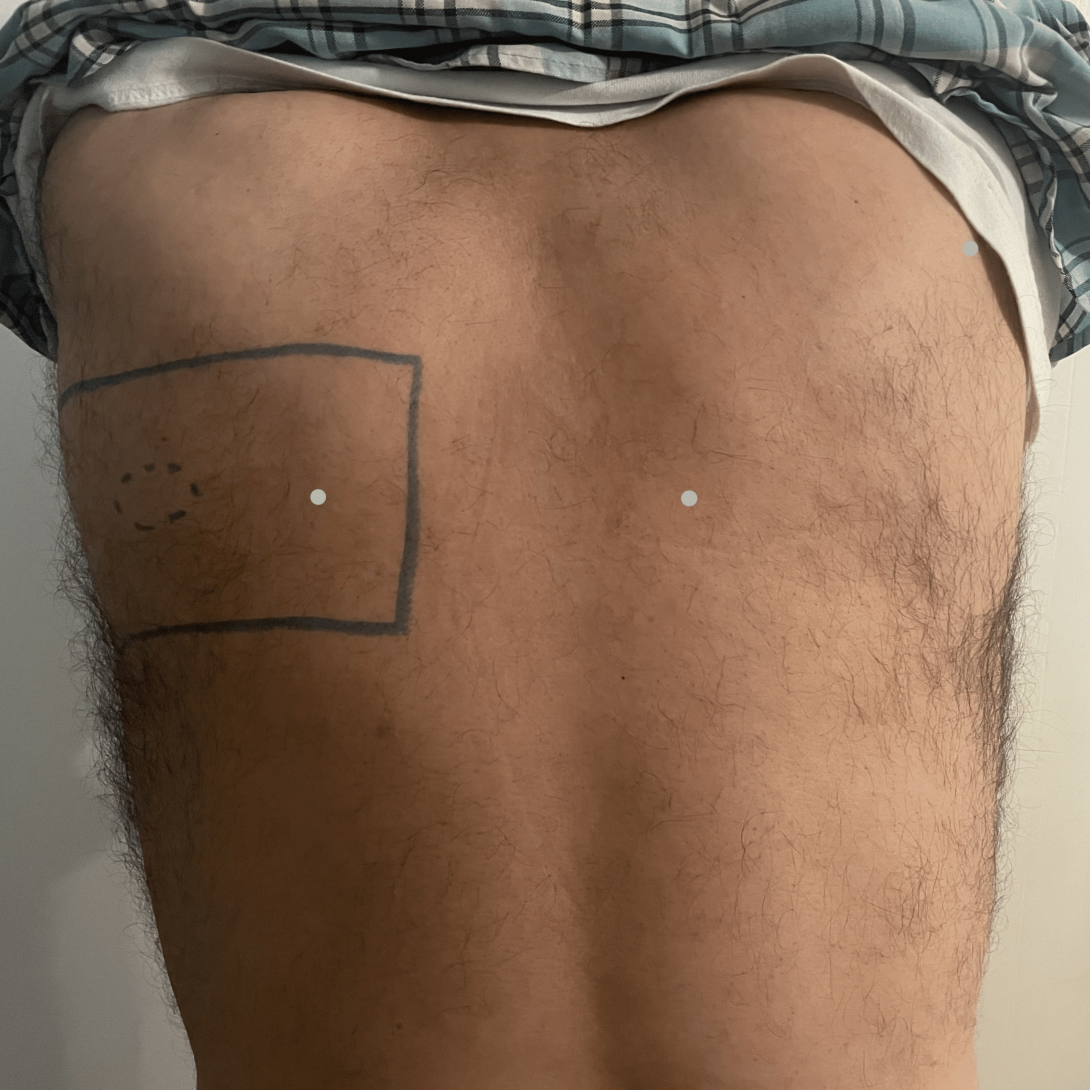
The patient's back showing the region of tenderness in the upper thoracic region

**Figure 2:**
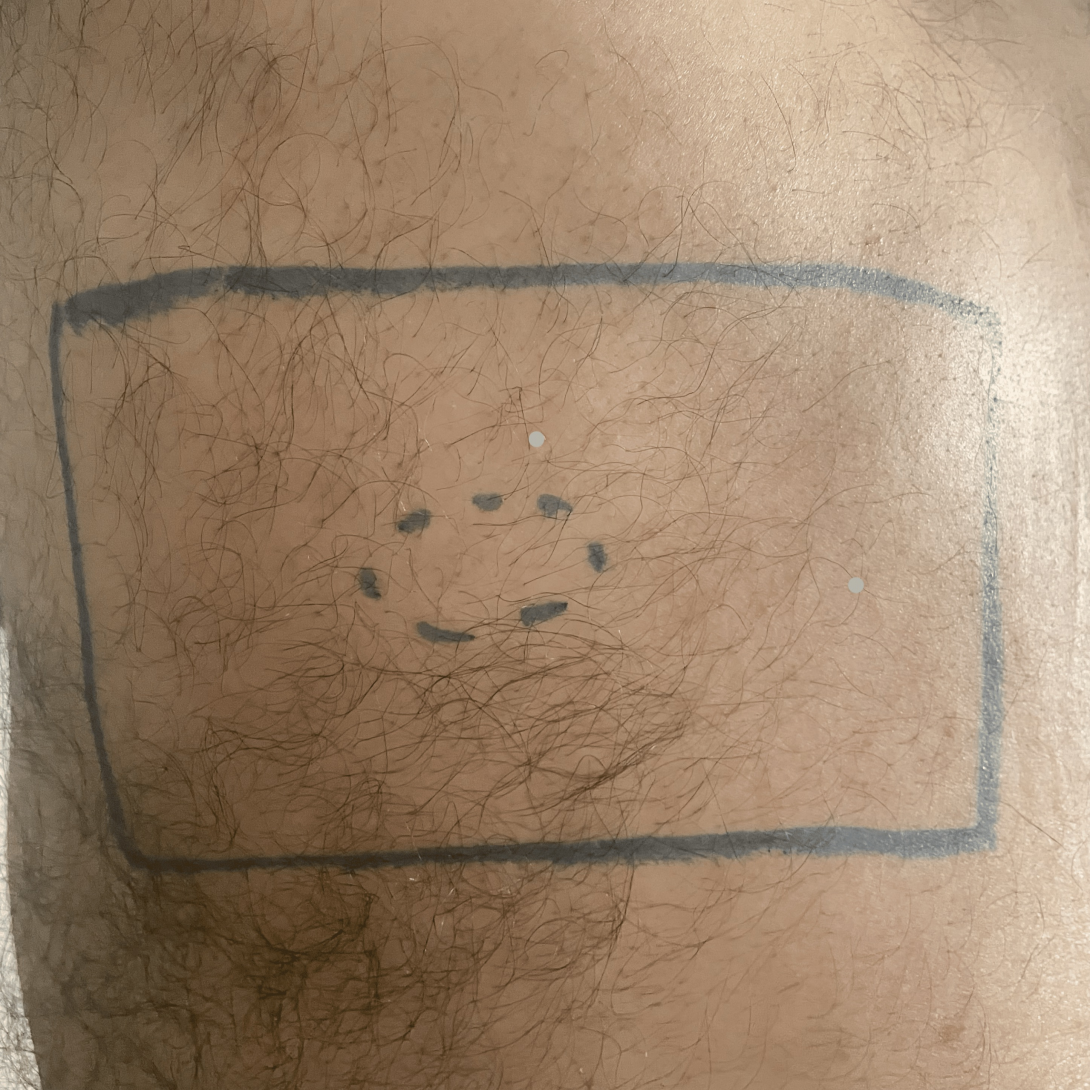
A photograph of the patient's back with a closer image highlighting the same region as in Figure 1

On examination, a faint pink patch was visualized over the left lateral upper back across the C4 and T2 dermatomes (Figures 1, 2).

Light touch of the lesion elicited an acute, sharp pain response. The patient's persistent burning, painful, and pruritic sensations, combined with a childhood diagnosis of chickenpox, were consistent with PHN. Initial treatment included amitriptyline 25 mg orally once daily, pregabalin 75 mg orally once daily, and a compounded topical cream containing amitriptyline 2%, baclofen 5%, ketamine 10%, and ketoprofen 10% applied twice daily, as well as lidocaine 5% topical patches as needed. Gabapentin treatment was discontinued.

At the one-month follow-up, the patient reported a minor improvement in pain with the current treatment regimen but continued to experience irritation during daily activities, particularly when driving. Consequently, the compounded topical cream (amitriptyline 2%/baclofen 5%/ketamine 10%/ketoprofen 10%) was continued, and all other treatments were discontinued as the patient stated he did not find any symptom relief outside of applying the topical ointment over the affected area.

The patient then presented to the emergency room weeks after the initial dermatology visit for PHN, with a chief complaint of “the mouth isn’t working when drinking coffee” and right-sided facial numbness. He reported that the initial onset of symptoms had occurred two days prior. Vital signs were recorded as follows: BP: 148/83 mmHg, pulse 74/min, respiratory rate 16/min, temperature 98.2 °F, and resting oxygen saturation: 98%. The physical exam was pertinent for facial asymmetry and drooping of the right side of the face. The patient was not in any pain or acute distress and denied experiencing chest pain, shortness of breath, or fatigue. He was admitted for monitoring and further testing, including an ECG, hemogram, comprehensive metabolic panel (CMP), and head and neck CT imaging.

All labs and imaging were negative for myocardial infarction (MI) and cerebrovascular accident (CVA). He was administered 325 mg of aspirin orally and 125 mg of intravenous methylprednisolone. A diagnosis of Bell’s palsy was made, and the patient was prescribed valacyclovir 500 mg, to be taken as two tablets orally three times daily for seven days. He later reported not following up with dermatology as his PHN and Bell’s palsy symptoms were well-controlled or negligible. He also noted no difficulty sleeping and denied needing to use topical or oral prescriptions to manage symptoms. Upon further communication with the patient, he reported that his symptoms had resolved completely, and he no longer had any chronic manifestations of his initial PHN and secondary Bell's palsy presentation. 

## Discussion

HZ arises from the reactivation of the VZV, which remains dormant in the sensory ganglia following an initial infection. Upon reactivation, VZV travels from the ganglia to the epidermis, resulting in a painful, maculopapular rash that typically presents unilaterally in a single dermatome. There is growing evidence linking HZ to Bell's palsy, a condition that accounts for up to 75% of peripheral facial paralysis cases [7]. It is postulated that VZV may reside in the geniculate ganglion of cranial nerve VII, and upon reactivation, leads to Bell’s palsy. When Bell’s palsy occurs without an obvious herpetiform rash, it is referred to as zoster sine herpete, which best represents the given case.

Given our patient's history of childhood chickenpox, clinical presentation, and the absence of a shingles vaccine, VZV reactivation and the subsequent development of PHN emerged as a strong differential diagnosis. This consideration was further supported by his type 2 diabetes mellitus, a known risk factor for both HZ and PHN [8]. Ultimately, the onset of Bell's palsy symptoms-often linked to VZV reactivation-confirmed that the patient’s condition aligned closely with PHN. It is imperative to note that the subtle dermatological presentation within the C4 and T2 dermatomes was the only visual evidence of a potential VZV reactivation. Therefore, this is a clinical diagnosis, a conclusion based on a multisystem assessment of the patient’s presentation.

The estimated lifetime risk of developing HZ is approximately 33%. The incidence of PHN is estimated at 57.5 cases per 100,000 person-years [3,9]. Approximately 20% of HZ-diagnosed individuals aged 60-65 years progress to having PHN, a figure that escalates to over 30% in those over 80 years of age [8]. Among individuals aged over 50 and 80 years, approximately 18% and 33%, respectively, will develop PHN after the onset of HZ [10]. The prevalence of PHN is 12.5% of patients aged 50 years and older developing PHN within three months of HZ onset [11]. Other significant risk factors include prodromal pain, extensive rash, female sex, and compromised cell-mediated immunity [10,11].

Female sex and the risk of PHN vary by age, with females under 60 years old having a relatively lower risk compared to those over 60 [11]. The global incidence of HZ is reported at 3-5 cases per 1,000 person-years in North America and Europe, while the Asia-Pacific region shows a slightly higher incidence of 3-10 cases per 1,000 person-years [12]. The risk of developing PHN in the Asia-Pacific region ranges from 6.2% to 52.0%, compared to 5.0% to 20.0% in the United States [3,12]. In the Asia-Pacific region, the most common comorbidities associated with PHN are hypertension (35.7%) and diabetes (18.1%) [12].

While the shingles vaccine is over 90% effective in preventing HZ and PHN and is considered the first line for prophylaxis, Fukushima et al. discuss using valacyclovir in the case of immunosuppression [13]. Valacyclovir is well-tolerated and can be administered at doses that maintain high plasma concentrations, enhancing its antiviral activity compared to acyclovir [14]. Valacyclovir provides an effective treatment option with a low recurrence rate of VZV reactivation and good tolerance among patients [13]. Valacyclovir has proven to aid in maintaining the patient’s well-controlled status in this case. 

PHN is often underdiagnosed in primary care due to its nonspecific symptoms, particularly chronic pain [6]. Delay of appropriate management of PHN may lead to additional complications such as depression, appetite loss, and sleep disturbances [8]. Prior dermatomal rash with blisters and persistent paresthesias are key indicators for PHN. Physical examination may reveal allodynia, altered sensation, and cutaneous scarring. Laboratory testing for VZV DNA and MRI can aid in distinguishing herpes zoster from herpes simplex and detecting HZ-induced lesions, respectively.

The simultaneous onset of PHN and Bell’s palsy in this patient represents an unusual clinical presentation, particularly in the context of varicella-zoster virus (VZV) reactivation. While both conditions can independently arise following VZV reactivation, their concurrent appearance is rare. Typically, PHN develops secondary to inflammatory damage in central and peripheral neurons following a painful, vesicular shingles rash [15]. Bell’s palsy, generally idiopathic, has been associated with viral infections affecting the facial nerve; evidence indicates that VZV can reactivate in the geniculate ganglion, contributing to peripheral facial paralysis in some cases [16]. The simultaneous development of these conditions may suggest a more extensive VZV reactivation involving multiple neural regions.

The absence of a prior shingles rash in this patient adds a diagnostic challenge, as VZV reactivation classically manifests with cutaneous lesions, facilitating early recognition [17]. Furthermore, the patient’s presentation of bilateral shoulder paresthesias suggests a broader neuropathic process, deviating from the typically localized nerve involvement seen in PHN [18]. This constellation of findings highlights the importance of considering VZV-related etiologies, even in the absence of hallmark clinical features [19].

The standard treatment for postherpetic neuralgia (PHN) typically includes first-line pharmacological options like tricyclic antidepressants (e.g., amitriptyline), gabapentinoids (e.g., pregabalin, gabapentin), and topical lidocaine patches [20]. For patients who do not respond adequately, second-line treatments may involve opioid analgesics or topical capsaicin [20]. In refractory cases, interventional options such as nerve blocks or transcutaneous electrical nerve stimulation (TENS) are considered [21].

In our case, the treatment protocol deviated from the usual approach. Initially, gabapentin and lidocaine patches were prescribed, but they failed to provide significant relief. The patient was then switched to a more tailored regimen, including amitriptyline, pregabalin, and a compounded topical cream containing amitriptyline, baclofen, ketamine, and ketoprofen. This personalized approach aimed to address the patient's specific symptoms, such as burning and pruritus. After one month, while the pain showed minor improvement, the compounded cream became the primary treatment, and other medications were discontinued to better manage his symptoms and improve quality of life.

Prevention strategies should focus on identifying high-risk populations and promoting vaccination, while early intervention in HZ cases can help prevent the progression to PHN [8]. Once PHN symptoms are established, the condition may become refractory to treatment, underscoring the importance of prevention.

## Conclusions

This report highlights the complexities of managing PHN in a patient with a history of type 2 diabetes and VZV reactivation. The patient's symptoms ultimately aligned with PHN, underscored by the onset of Bell's palsy. The successful use of a compounded topical therapy emphasizes the importance of early intervention and a tailored approach in PHN management. Further research is needed to improve our understanding of the factors influencing PHN risk and to optimize diagnostic and treatment strategies for this challenging condition.

## References

[1] Niemeyer CS, Harlander-Locke M, Bubak AN, Rzasa-Lynn R, Birlea M (2024). Trigeminal postherpetic neuralgia: from pathophysiology to treatment. Curr Pain Headache Rep.

[2] Aggarwal A, Suresh V, Gupta B, Sonthalia S (2020). Post-herpetic neuralgia: a systematic review of current interventional pain management strategies. J Cutan Aesthet Surg.

[3] Mallick-Searle T, Snodgrass B, Brant JM (2016). Postherpetic neuralgia: epidemiology, pathophysiology, and pain management pharmacology. J Multidiscip Healthc.

[4] Roybal AE, Sivanesan E, Chen Y (2021). Case report: dorsal root ganglion (DRG) stimulation for acute neuropathic pain from acute herpes zoster infection. SAGE Open Med Case Rep.

[5] Mercan A, Uzun ST, Keles S, Hacibeyoglu G, Yilmaz R, Reisli R (2021). Immunological mechanism of postherpetic neuralgia and effect of pregabalin treatment on the mechanism: a prospective single-arm observational study. Korean J Pain.

[6] Nalamachu S, Morley-Forster P (2012). Diagnosing and managing postherpetic neuralgia. Drugs Aging.

[7] Freire de Castro R, Crema D, Neiva FC, Pinto RA, Suzuki FA (2022). Prevalence of herpes zoster virus reactivation in patients diagnosed with Bell's palsy. J Laryngol Otol.

[8] Gruver C, Guthmiller KB (2025). Postherpetic Neuralgia. https://pubmed.ncbi.nlm.nih.gov/29630250/.

[9] Thompson RR, Kong CL, Porco TC, Kim E, Ebert CD, Acharya NR (2021). Herpes zoster and postherpetic neuralgia: changing incidence rates from 1994 to 2018 in the United States. Clin Infect Dis.

[10] Chae JS, Im J, Choi YJ, Lee HJ, Kim WJ (2023). Comparison of the severity of zoster-associated pain and incidence of postherpetic neuralgia in patients with and without pre-existing spinal disorders at the same spinal nerve level: a retrospective multicenter study. J Pers Med.

[11] Forbes HJ, Thomas SL, Smeeth L, Clayton T, Farmer R, Bhaskaran K, Langan SM (2016). A systematic review and meta-analysis of risk factors for postherpetic neuralgia. Pain.

[12] Yang F, Yu S, Fan B (2019). The epidemiology of herpes zoster and postherpetic neuralgia in China: results from a cross-sectional study. Pain Ther.

[13] Fukushima T, Sato T, Nakamura T (2012). Daily 500 mg valacyclovir is effective for prevention of varicella zoster virus reactivation in patients with multiple myeloma treated with bortezomib. Anticancer Res.

[14] Fei N, Shah N, Cumpston A, Wen S, Ross KG, Craig M, Kanate AS (2019). Low-dose acyclovir prophylaxis for varicella zoster reactivation in autologous hematopoietic cell transplantation recipients. Clin Hematol Int.

[15] Gilden DH, Kleinschmidt-DeMasters BK, LaGuardia JJ, Mahalingam R, Cohrs RJ (2000). Neurologic complications of the reactivation of varicella-zoster virus. N Engl J Med.

[16] Crouch AE, Hohman MH, Moody MP, Andaloro C (2023). Ramsay Hunt Syndrome. Stat Pearls.

[17] Nakamura Y, Miyagawa F, Okazaki A (2016). Clinical and immunologic features of recurrent herpes zoster (HZ). J Am Acad Dermatol.

[18] Watson PNC, Evans RJ, Watt VR, Birkett N (1988). Post-herpetic neuralgia: 208 cases. Pain.

[19] Yang Y, Mahmood T, Siddiqui AH, Aziz MA (2023). Zoster Sine Herpete: two unusual cases of varicella-zoster reactivation with atypical complaints of acute chest pain and severe headache. BMC Infect Dis.

[20] Gan EY, Tian EA, Tey HL (2013). Management of herpes zoster and post-herpetic neuralgia. Am J Clin Dermatol.

[21] Lin CS, Lin YC, Lao HC, Chen CC (2019). Interventional treatments for postherpetic neuralgia: a systematic review. Pain Physician.

